# Involvement of the Ubiquitin-Proteasome System in the Formation of Experimental Postsurgical Peritoneal Adhesions

**DOI:** 10.1155/2012/194723

**Published:** 2012-02-14

**Authors:** Clara Di Filippo, Pasquale Petronella, Fulvio Freda, Marco Scorzelli, Marco Ferretti, Sivestro Canonico, Francesco Rossi, Michele D'Amico

**Affiliations:** ^1^Department of Experimental Medicine, Section of Pharmacology “L. Donatelli”, Second University of Naples, 80138 Naples, Italy; ^2^Department of Gerontology, Geriatric and Metabolic Diseases, Second University of Naples, 80138 Naples, Italy

## Abstract

We investigated the Ubiquitin-Proteasome System (UPS), major nonlysosomal intracellular protein degradation system, in the genesis of experimental postsurgical peritoneal adhesions. We assayed the levels of UPS within the adhered tissue along with the development of peritoneal adhesions and used the specific UPS inhibitor bortezomib in order to assess the effect of the UPS blockade on the peritoneal adhesions. We found a number of severe postsurgical peritoneal adhesions at day 5 after surgery increasing until day 10. In the adhered tissue an increased values of ubiquitin and the 20S proteasome subunit, NFkB, IL-6, TNF-**α** and decreased values of IkB-beta were found. In contrast, bortezomib-treated rats showed a decreased number of peritoneal adhesions, decreased values of ubiquitin and the 20S proteasome, NFkB, IL-6, TNF-**α**, and increased levels of IkB-beta in the adhered peritoneal tissue. The UPS system, therefore, is primarily involved in the formation of post-surgical peritoneal adhesions in rats.

## 1. Introduction

Postsurgical adhesions are polymorphic clinical pictures ranging from abdominal and pelvic pain to intestinal mechanical occlusion. They are due to stable lesions scarring, the adhesions. Intraperitoneal adhesions are connective bridges between adjacent portions of the peritoneum [1], their formation occurs in approximately 90% of patients undergoing abdominal surgery, and it is an important reason of post-operative morbidity and mortality [2].

Adhesions formation is initiated by the trauma surgery, and it is supported by a number of factors including fibrin, thromboplastin, mesothelial cells, fibroblasts, collagen fibrils, and the development of a local inflammatory process, among others [3].

Until a few years ago to prevent the formation of adhesions, the focus was mainly on the procedures to be implemented during surgery. It is worth mentioning that there is considerable consensus that laparoscopic surgery is associated with the reduced development of adhesions compared to open surgery in the international arena [4, 5].

A surgical trauma stimulates the release of several chemical mediators of inflammation, including IL-1, IL-6, and TNF-*α* whose involvement in the inflammatory process, that underlies the formation of adhesions, has been documented in many studies [6].

The transforming growth factor beta-1 (TGF-*β*1) and transforming growth factor beta-3 (TGF-*β*3) appear to be involved in the process too. They are released after hypoxia generated by tissue injury during surgery [7]. 

In recent years numerous studies have been aimed at a likely pharmacological prevention of postsurgical adhesion formation. For example, some authors proposed the use of statins as they are considered as being able to increase the peritoneal fibrinolysis [8]. Other authors have suggested that interferon gamma is a possible therapeutic target to prevent the formation of adhesions, due to its crucial role in the differential regulation of PAI-1 and t-PA, which are involved in this process [9]. However other ones suggest the use of antitack agents such as carboxymethylcellulose and hyaluronic acid resorbable membranes. Finally it is also alleged the possible use of propofol [10].

In this wide scenario of hypotheses we want to include the centrality of the inflammatory component in the unfolding process of adhesions; we hypothesized that the Ubiquitin-Proteasome System (UPS), the major nonlysosomal intracellular protein degradation system in eukaryotic cells and which is capable of inducing inflammation [11–14], may play a major role in the formation of post-surgical peritoneal adhesions. We hypothesized that the activation of this system during the surgery can be suggestive of the beginning of inflammatory events that in cascade lead to adhesions formation. So, in this experimental study on rats we have evaluated whether changes in UPS levels and activity into the adhered tissue occur along with the development of post-surgical peritoneal adhesions, and then we used the specific UPS inhibitor bortezomib [15–17] in order to ascertain the role that the UPS may have in the peritoneal adhesions formation.

## 2. Methods

### 2.1. Surgical Procedure

All experimental procedures and protocols used in this study were reviewed and approved by the Special Ethics Commission at the 2nd University of Naples.

The surgery was performed as previously described [4]. Male Sprague-Dawley rats (*n* = 15) were marked with a pencil as 1 to 15, anesthetized with urethane (1.2 g/kg ip), subjected to midline laparotomy. A sample of parietal peritoneal tissue was taken and an enterotomy was performed at the level of the ileum. The surgical incision was sutured with absorbable surgical wire 4/0 in order to induce an inflammatory peritoneal insult. All of the surgical procedure was then ended by a nonabsorbable suture, and the rats were placed in the recovering room for awakening. Five days after the surgery the rats were subjected to another laparotomy, had a new tissue sample taken, and were assayed for the development of peritoneal adhesions by means of qualitative and quantitative evaluation. A score from 1 to 6 was established, and it was given as follows: 1 to the presence of poor and lapse adhesions in a limited peritoneal zone; 2 to the presence of poor adhesions in an extended zone; 3 to the presence of several lapse adhesions into the peritoneum; 4 to the presence of localized dense adhesions; 5 to the presence of extended dense adhesions with access to peritoneal cavity; 6 to the presence of extended dense adhesions and impossible access to peritoneal cavity. The same procedure was repeated after 10 days from the first surgery, having particular attention to keep always the same rat numbering over the time course considered. A surgeon blinded for the procedure and different from the one who performed the surgery scored the adhesion. Biopsies of peritoneal tissue were snap-frozen and used to determine: TNF-alpha, IL6, NFkB, and activity of the UPS system.

#### 2.1.1. Experimental Groups

The study was conducted on male Sprague-Dawley rats (4-month old and weighing 200 g) induced with only the surgical procedure (*n* = 15) or treated with bortezomib + surgical procedure (*n* = 15). This latter was first treated with bortezomib (Velcade; Millennium Pharmaceuticals, Cambridge, UK) by intravenous injection (0.05 mg/kg) given 1 hour before surgery, and a second injection was given 1 hour after the surgical procedure (total dose of 0.1 mg/kg) according to our previous experience in other setting [4].

There were no significant side effects after injections.

#### 2.1.2. Biochemical Parameters Assessed

The tissue samples taken during the surgery were homogenized in 50 mM Tris-HCl (pH 7.2) containing leptin 1 *μ*M, pepsatin A 1 *μ*M, and phenyl methyl sulfonyl fluoride 200 *μ*M and centrifuged for 10 min at 10,000 ×g at 4°C.

200 *μ*L of homogenate were used to determine total protein according to the Bradford's method. The levels of ubiquitin, IL-6, and TNF-alpha were quantified by a commercial colorimetric ELISA kit (R&D Systems, USA). For the quantitative measurement of the 20S proteasome, a specific SDS activation kit (Boston Biochem, USA) was used following the instructions of the manufacturer.

#### 2.1.3. Statistics

Data are presented as mean ± SE. Continuous variables were compared among the groups of rats with one-way analysis of variance (ANOVA) for normally distributed data and Kruskal-Wallis test for nonnormally distributed data. When differences were found among the groups, Bonferroni correction was used to make pairwise comparisons. A *P* < 0.05 was considered statistically significant. All calculations were performed using the SPSS2 software.

## 3. Results

**Figure 1:**
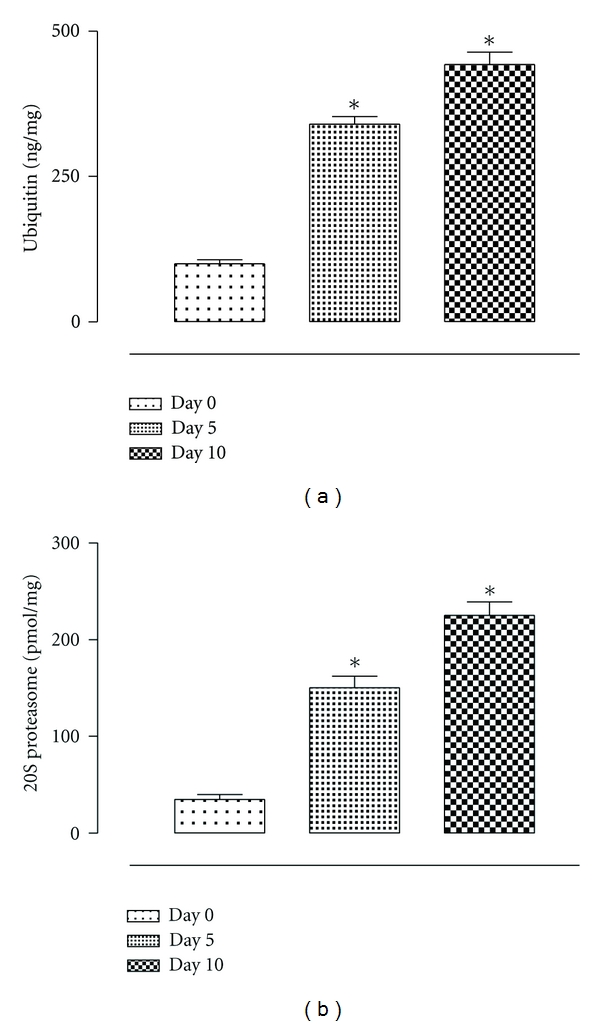
Levels of ubiquitin and 20S proteasome in the tissues from rats during peritoneal adhesions development at time 0, 5, and 10 days from the surgery in the control group. The differences from day 0 are considered with *P < 0.01.

Starting from day 5 from surgery there was a development of peritoneal adhesions within the rats. After 10 days from the surgery 7 control rats were scored 6 for the severity of the adhesions, 4 rats were scored 5, and 4 control rats were scored 1 for the presence of poor and lapse adhesions in a limited peritoneal zone. An increase in the levels of ubiquitin and the 20S proteasome within the adhered tissue was observed in parallel with the development of peritoneal adhesions. For both the markers this increase was significant (*P* < 0.01 versus day 0) already 5 days after surgery (Figure 1). A further increase in the levels of ubiquitin and the 20S proteasome was observed in the adhered peritoneal tissue after 10 days of surgery (*P* < 0.01 versus day 0) (Figure 1).

**Figure 2:**
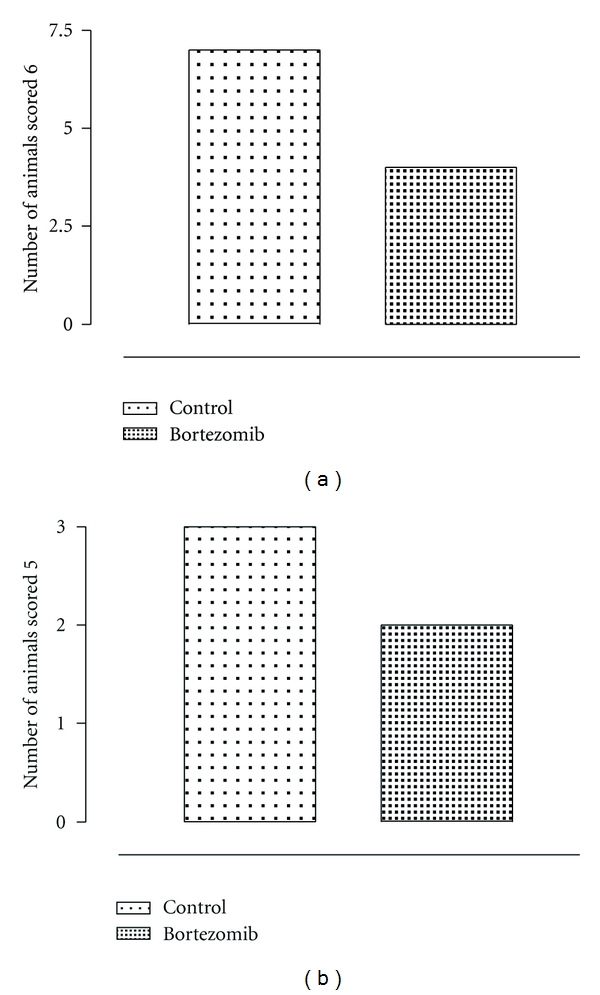
Quantitative evaluation by the peritoneal adhesions score indicating a substantial decrease of adhesions in the group of rats treated with bortezomib after 10 days of surgery if compared with the control group same day.

**Figure 3:**
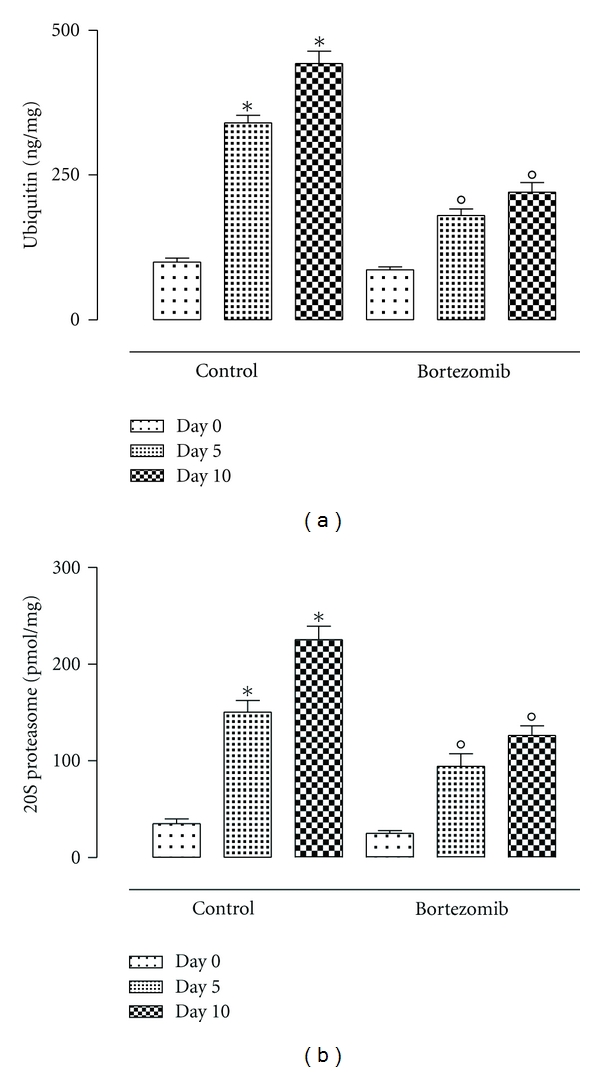
Levels (ng/mg tissue) of ubiquitin and 20S proteasome in the tissues from rats during peritoneal adhesions development. At time 0, 5, and 10 in the group treated with bortezomib by intravenous injection (0.05 mg/kg) given 1 hour before surgery, a second injection was given 1 hour after the surgical procedure (total dose of 0.1 mg/kg) and in the control group. The differences from day 0 are considered with *P < 0.01, and the differences from the control group at the same day are considered as °*P* < 0.01.

**Figure 4:**
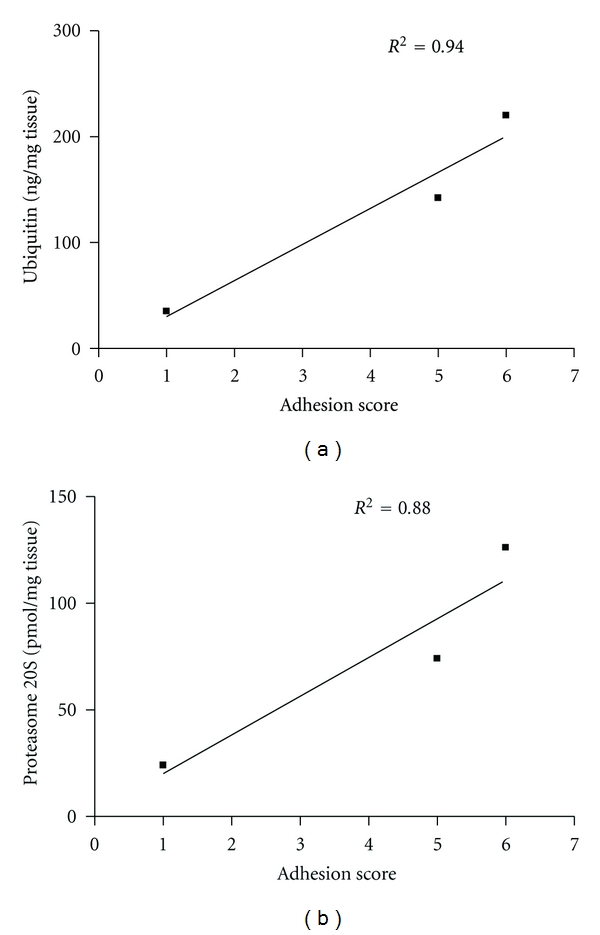
Correlation between the adhesion score and the local levels of ubiquitin and protesome 20S 10 days after the surgery.

The qualitative evaluation by the peritoneal adhesions score indicates a substantial decrease of adhesions in the group of rats treated with bortezomib (e.g., 4 rats were scored 6 and 2 rats were scored 5) after 10 days of surgery if compared with the control group same day (Figure 2). This was accompinied by a significant reduction of the levels of both ubiquitin and proteasome 20S (*P* < 0.01 versus day 0) in the peritoneal tissue 5 days after surgery. The reduction was found to be significant even 10 days after surgical procedure (*P* < 0.01 versus day 0) (Figure 3). At this time point there was a significant correlation between the levels of ubiquitin/proteasome 20S and the adhesion score assigned to the rats after bortezomib treatment (Figure 4).

**Figure 5:**
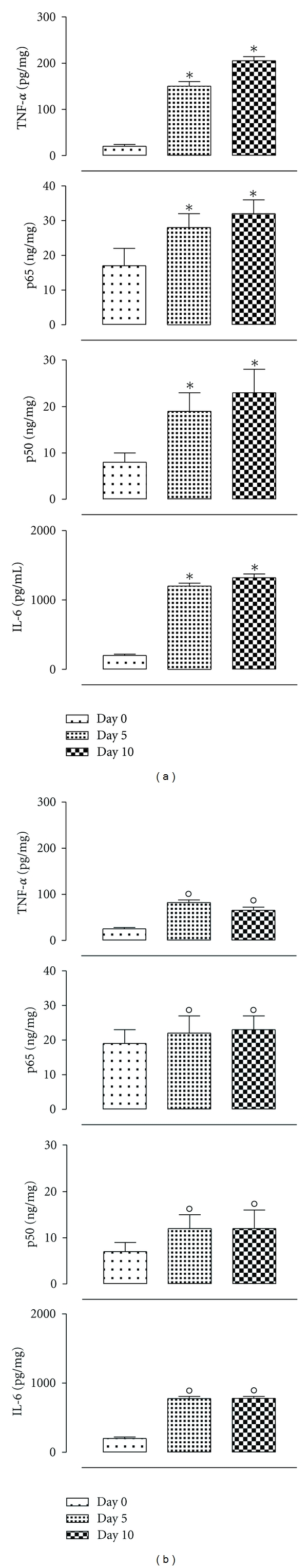
Levels (ng/mg tissue) of TNF-alpha, p50, p65, and IL-6 within the tissues from rats during peritoneal adhesions development at time 0, 5, and 10 days from the peritoneal surgery. The rats were treated or not with bortezomib by intravenous injection (0.05 mg/kg) given 1 hour before surgery, and a second injection was given 1 hour after the surgical procedure (total dose of 0.1 mg/kg). The differences from day 0 are considered with *P < 0.01, and the differences from the control group at the same day are considered as °*P* < 0.01.

**Figure 6:**
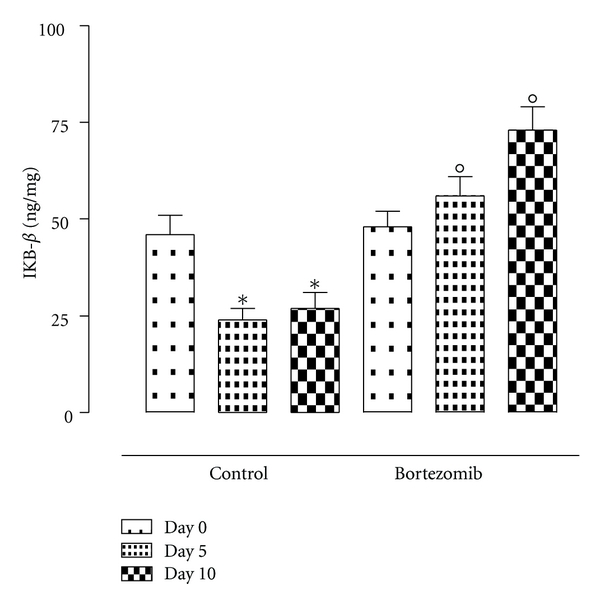
IKB-*β* levels (ng/mg tissue) within the tissues from rats during peritoneal adhesions development. Rats at time 0, 5, and 10 days from the surgery and treated or not with bortezomib. The differences from day 0 are considered with *P < 0.01, and the differences from the control group at the same day are considered as °*P* < 0.01.

In the rat adhered peritoneal tissue the TNF-alpha, IL-6, p50, and p65 (subunit of NFkB) levels were significantly increased after 5 and 10 days after surgery (Figure 5). In contrast, in the same tissue the levels of the IkB-beta both at day 5 and at day 10 (*P* < 0.01 versus day 0) were seen decreased (Figure 6).

Treatment with bortezomib resulted in reduced levels of TNF-alpha and IL-6, p50, and p65 in both time day 5 and time day 10 (*P* < 0.01 versus control group with adhesions) (Figure 5), together with a significant increase in levels of IkB-beta (*P* < 0.01 versus control group) (Figure 6).

## 4. Discussion

In a previous research this group found that post-surgical peritoneal adhesions occur in rats when they are subject to laparatomy and enterotomy of the ileum [4]. These adhesions are strictly related to the time from the surgery and have severity depending on the inflammatory response occurring within the peritoneal specimens, which deteriors the peritoneal matrix. During this phase, leukocytes, cytokines, chemokines, and cell adhesion molecules alters the initial tissutal equilibrium predisposing them to the formation of adhesions [4].

In the present study it is shown that a key role in the tissutal alterations that follows a surgical intervention and development of peritoneal adhesions is assumed by the Ubiquitin-Proteasome System (UPS). This study demonstrates that the activity of this system, measured on biopsies of the adhered tissue, relates to the severity of the adhesion; animals that show high levels of UPS activity also have severe adhesions, whilst animals that show low levels of UPS have moderate adhesions.

UPS, generally known as the major pathway for nonlysosomal intracellular protein degradation in eukaryotic cells, was discovered in the eighties by the pioneering work of Goldberg, Hershko, and their collaborators, using reticulocyte lysates [18–20]. The UPS usually degrade proteins in two steps. First, the substrate is covalently modified by addition of a polyubiquitin chain, through an enzymatic cascade that involves three classes of factors: E1, the ubiquitin-(Ub-)activating enzyme [21], E2, a member of the family of Ub-carriers [22], and E3, a member of the very large family (several hundred members) of the so-called Ub-ligases [23], which specifically recognizes and recruits the substrate of the ubiquitylation reaction. Second, the ubiquitylated protein is usually addressed to and degraded by the 26S proteasome, a giant multisubunit and multicatalytic protease [24]. Due to its multiple roles, the proteasome is essential in eukaryotes and its dysfunction has deleterious effects for the cell or the organism as a whole. In humans, UPS deregulation has been implicated in a number of pathologies such as cancer, autoimmune diseases, neurodegenerative diseases, or viral infections. As a consequence, the proteasome is seen as a potential therapeutic target in many pathologies [25], including inflammation [26] and possibly now peritoneal adhesions.

In the present study is also shown that post-operative adhesions are associated with decreased levels of the inhibitory protein for the transcriptor factor nuclear factor kappa B (NF-kB), the IkB within the adhered tissues, indicating that one other component in the generation of adhesion is the activated NF-kB pathway, a central transcription factor that regulates inflammatory genes. Activated NF-kB pathway leads tissue deregulation and favours cell proliferation in many circumstances [27–29]. Interestingly, the UPS is required for activation of NF-kB by degradation of its inhibitory IkB proteins [30]. NFkB is normally bound to IkB in the cytosol; this binding prevents its movement into the nucleus [31]. Various cellular stimuli, such as inflammatory stimuli, induce ubiquitination of phosphorylated IkBs and subsequent degradation by the proteasome [32, 33]. Degradation of IkBs results in unmasking of the nuclear localization signal of NF-kB dimers, which subsequently translocates to the nucleus, where it induces the transcription of proinflammatory cytokines like IL-6 and TNF-*α* that play a central role in tissue injury [34]. Here we would suggest that a surgical insult may induce degradation of IkBs via ubiquitin-proteasome overactivity, thus enhancing NFkB activation. This in turn enhances the inflammatory potential of the injured tissue, worsening the peritoneal recovery. A contention is based on the fact that the levels of UPS, IkB, TNF-alpha, and IL-6 measured on adhesions biopsies seem to be related to the severity of the adhesion. Animals that show moderate levels of UPS, TNF-alpha, and IL-6 have also less severe adhesions than those with higher levels of these same mediators.

Proof of concept for a central role of UPS in peritoneal adhesions is given by the protection from the adhesion formation achieved with the treatment of injured rats with bortezomib. This drug, initially proposed as anticancer drug [35], is a specific inhibitor of the UPS activity and exerts inhibition of the inflammation associated to the transcription factor NF*κ*B, through stabilization of its inhibitor I*κ*B [36], since NF*κ*B is bound to its inhibitor I*κ*B in the cytoplasm and is activated by proteasomal degradation of I*κ*B. Inhibition of proteasome activity prevents degradation of I*κ*B and subsequent activation and translocation of NF*κ*B to the nucleus to activate downstream pathways [36]. Bortezomib here is able to reduce the number of rats showing adhesions and the score assigned to the adhesions, and as consequence of UPS inhibition, bortezomib also reduced the burden of inflammation occurring in the adhered tissue. At the same time point, in fact, rats treated with bortezomib had the lowest level of ubiquitin and proteasome 20S activity, NF-kB activation, inflammatory cells, and cytokine levels associated with the reduced adhesions formation.

In conclusion, post-surgical peritoneal adhesions formation is associated with local increased UPS system activity which is primarily involved in the formation of post-surgical peritoneal adhesions in rats and with a local inflammatory factors boost.

## References

[1] Canis M, Botchorishvili R, Wattiez A (2001). Prevention of peritoneal adherences. *Journal de Gynecologie Obstetrique et Biologie de la Reproduction*.

[2] Attard JAP, Maclean AR (2007). Adhesive small bowel obstruction: epidemiology, biology and prevention. *Canadian Journal of Surgery*.

[3] Ott DE (2008). Laparoscopy and adhesion formation, adhesions and laparoscopy. *Seminars in Reproductive Medicine*.

[4] Di Filippo C, Falsetto A, De Pascale V (2006). Plasma levels of t-PA and PAI-1 correlate with the formation of experimental post-surgical peritoneal adhesions. *Mediators of Inflammation*.

[5] Lauder CIW, Garcea G, Strickland A, Maddern GJ (2010). Abdominal adhesion prevention: still a sticky subject?. *Digestive Surgery*.

[6] Schnriger B, Barmparas G, Branco BC, Lustenberger T, Inaba K, Demetriades D (2011). Prevention of postoperative peritoneal adhesions: a review of the literature. *American Journal of Surgery*.

[7] Holmdahl L, Ivarsson ML (1999). The role of cytokines, coagulation, and fibrinolysis in peritoneal tissue repair. *European Journal of Surgery*.

[8] Suzuki N, Imai A (2010). HMG-CoA reductase inhibitor lovastatin causes reversible cytoskeleton perturbation by RhoA signalling suppression in peritoneal cell line Met5A. *Journal of Obstetrics and Gynaecology*.

[9] Tietze L, Elbrecht A, Schauerte C (1998). Modulation of pro- and antifibrinolytic properties of human peritoneal mesothelial cells by transforming growth factor *β*1 (TGF-*β*1), tumor necrosis factor *α* (TNF-*α*) and interleukin 1*β* (IL-1*β*). *Thrombosis and Haemostasis*.

[10] Aykas A, Yuzbasioglu MF, Kurutas EB (2010). Protective effects of propofol on peritoneal adhesions in cecal ligation and puncture model. *Bratislavské Lekárske Listy*.

[11] Marfella R, Filippo CD, Portoghese M (2009). The ubiquitin-proteasome system contributes to the inflammatory injury in ischemic diabetic myocardium: the role of glycemic control. *Cardiovascular Pathology*.

[12] Di Filippo C, Marfella R, D’Amico M (2008). Possible dual role of ubiquitin-proteasome system in the atherosclerotic plaque progression. *Journal of the American College of Cardiology*.

[13] Marfella R, D’Amico M, Esposito K (2006). The ubiquitin-proteasome system and inflammatory activity in diabetic atherosclerotic plaques: effects of rosiglitazone treatment. *Diabetes*.

[14] Marfella R, Filippo CD, Portoghese M (2009). The ubiquitin-proteasome system contributes to the inflammatory injury in ischemic diabetic myocardium: the role of glycemic control. *Cardiovascular Pathology*.

[15] Van Herck JL, De Meyer GRY, Martinet W, Bult H, Vrints CJ, Herman AG (2010). Proteasome inhibitor bortezomib promotes a rupture-prone plaque phenotype in ApoE-deficient mice. *Basic Research in Cardiology*.

[16] Eldridge AG, O’Brien T (2010). Therapeutic strategies within the ubiquitin proteasome system. *Cell Death and Differentiation*.

[17] Yamamura M, Hirai T, Yamaguchi Y (2010). Proteasome inhibitor. *Nippon Rinsho*.

[18] Etlinger JD, Goldberg AL (1977). A soluble ATP dependent proteolytic system responsible for the degradation of abnormal proteins in reticulocytes. *Proceedings of the National Academy of Sciences of the United States of America*.

[19] Ciechanover A, Heller H, Elias S, Haas AL, Hershko A (1980). ATP-dependent conjugation of reticulocyte proteins with the polypeptide required for protein degradation. *Proceedings of the National Academy of Sciences of the United States of America*.

[20] Hershko A, Ciechanover A, Heller H, Haas AL, Rose IA (1980). Proposed role of ATP in protein breakdown: conjugation of protein with multiple chains of the polypeptide of ATP-dependent proteolysis. *Proceedings of the National Academy of Sciences of the United States of America*.

[21] Schulman BA, Wade Harper J (2009). Ubiquitin-like protein activation by E1 enzymes: the apex for downstream signalling pathways. *Nature Reviews Molecular Cell Biology*.

[22] Ye Y, Rape M (2009). Building ubiquitin chains: E2 enzymes at work. *Nature Reviews Molecular Cell Biology*.

[23] Robinson PA, Ardley HC (2004). Ubiquitin-protein ligases. *Journal of Cell Science*.

[24] Voges D, Zwickl P, Baumeister W (1999). The 26S proteasome: a molecular machine designed for controlled proteolysis. *Annual Review of Biochemistry*.

[25] Stone PH, Muller JE, Hartwell T (1989). The effect of diabetes mellitus on prognosis and serial left ventricular function after myocardial infarction: contribution of both coronary disease and diastolic left ventricular dysfunction to the adverse prognosis. *Journal of the American College of Cardiology*.

[26] Hershko A, Ciechanover A, Varshavsky A (2000). Basic medical research award. The ubiquitin system. *Nature Medicine*.

[27] Frank S, Seitz O, Schürmann C (2010). Wound healing in mice with high-fat diet- or ob gene-induced diabetes-obesity syndromes: a comparative study. *Experimental Diabetes Research*.

[28] Hanzu FA, Palomo M, Kalko SG (2011). Translational evidence of endothelial damage in obese individuals: inflammatory and prothrombotic responses. *Journal of Thrombosis and Haemostasis*.

[29] Kirovski G, Stevens AP, Czech B (2011). Down-regulation of methylthioadenosine phosphorylase (MTAP) induces progression of hepatocellular carcinoma via accumulation of 5′-deoxy- 5′-methylthioadenosine (MTA). *American Journal of Pathology*.

[30] Palombella VJ, Rando OJ, Goldberg AL, Maniatis T (1994). The ubiquitin-proteasome pathway is required for processing the NF-*κ*B1 precursor protein and the activation of NF-*κ*B. *Cell*.

[31] Siebenlist U, Franzoso G, Brown K (1994). Structure, regulation and function of NF-*κ*B. *Annual Review of Cell Biology*.

[32] Hermann J, Gulati R, Napoli C (2003). Oxidative stress-related increase in ubiquitination in early coronary atherogenesis. *The FASEB Journal*.

[33] Herrmann J, Ciechanover A, Lerman LO, Lerman A (2004). The ubiquitin-proteasome system in cardiovascular diseases—a hypothesis extended. *Cardiovascular Research*.

[34] Sun M, Dawood F, Wen WH (2004). Excessive tumor necrosis factor activation after infarction contributes to susceptibility of myocardial rupture and left ventricular dysfunction. *Circulation*.

[35] Adams J, Palombella VJ, Sausville EA (1999). Proteasome inhibitors: a novel class of potent and effective antitumor agents. *Cancer Research*.

[36] Crawford LJ, Walker B, Irvine AE (2011). Proteasome inhibitors in cancer therapy. *Journal of Cell Communication and Signaling*.

